# Increased beta-band salience network connectivity predicts 12-month longitudinal changes in depressive and anxiety symptom severity in young adults: a resting-state EEG study

**DOI:** 10.1007/s00406-026-02277-z

**Published:** 2026-06-01

**Authors:** Claudio Imperatori, Mauro Adenzato, Simone Messerotti Benvenuti, Elena De Rossi, Giorgia Allegrini, Carola Dell’Acqua, Giuseppe Alessio Carbone, Rita B. Ardito, Benedetto Farina

**Affiliations:** 1 Experimental and Applied Psychology Laboratory, Department of Health and Life Sciences, European University of Rome, Rome, Italy; 2 Department of Psychology, University of Turin, Turin, Italy; 3 Department of General Psychology, University of Padua, Padua, Italy; 4 Padova Neuroscience Center (PNC), University of Padua, Padua, Italy; 5 Faculty of Psychology and Educational Sciences, University of Geneva, 40, Boulevard du Pont-d’Arve, 1205 Geneva, Switzerland

**Keywords:** Depression, Anxiety, Resting-state networks, EEG, eLORETA

## Abstract

The neural dynamics of large-scale brain networks that may be longitudinally associated with changes in depression and anxiety (DEPANX) symptom severity remain relatively understudied. We aimed to examine the relationship between the electrophysiological dynamics of the three-core resting-state networks (RSNs) and 12-month longitudinal changes in DEPANX symptom severity. Ninety-five participants (70 females; mean age: 21.35 ± 2.65 years) completed baseline (Time #1) and 12-month follow-up (Time #2) assessments. At Time #1 data about DEPANX symptoms and related variables (e.g., emotion regulation, familiarity for depression and anxiety) were collected. A resting-state electroencephalogram (EEG) recording was also collected. At Time #2 levels of DEPANX symptoms were assessed again. EEG data were analysed using the Exact Low-Resolution Electromagnetic Tomography software (eLORETA). We found that baseline SN connectivity, particularly bilateral insula beta coherence, was associated (*r* = 0.371; *p*_*adjusted*_ = 0.015) with DEPANX symptom changes over 12 months, whereas no comparable longitudinal associations were observed for other RSNs. When controlling for the presence of other variables, this connectivity pattern remained positively and independently associated with change in DEPANX symptoms (*β* = 0.328; *p* = 0.002). Our results suggest a key role of the bilateral insula connectivity in predicting 12-month longitudinal changes in DEPANX symptoms, with potential implications for prevention and treatment.

**Supplementary Information:**

The online version contains supplementary material available at 10.1007/s00406-026-02277-z.

## Introduction

Depression and anxiety symptoms are among the leading contributors to the global health burden and disability, particularly in young adults [1–3]. These conditions are widespread and highly comorbid. Recent meta-analyses report high rates of depressive (35.9%) and anxiety (40.7%) symptoms among college students [4] and a similar two- to three-fold bidirectional risk between depression and anxiety over time [5]. By 2040, the global burden of anxiety and depression is projected to increase significantly [3], highlighting the need for stronger prevention efforts and mental health interventions that primarily target the risk factors for these conditions [6].

Depression and anxiety symptoms are believed to result from a complex interplay of biopsychosocial factors [7, 8], many of which are common across these conditions. For example, a family history of depression and anxiety in first-degree relatives is considered one of the most important risk factors for developing these disorders [9–11]. Among environmental factors, exposure to adverse childhood experiences (ACEs), such as abuse and neglect, is recognized as a significant risk factor for both conditions [12, 13]. Depression and anxiety symptoms are also associated with other common risk factors, including female gender [14], maladaptive emotion regulation strategies [15], and substance misuse patterns [16], such as binge drinking [17]. Given their shared vulnerability mechanisms e.g., [18, 19] and high comorbidity [5], we conceptualized depression and anxiety symptoms as a single internalizing dimension (hereafter, DEPANX). This transdiagnostic approach aligns with prevention-oriented research in non-clinical samples, where mixed anxious–depressive symptom presentations are common and clinically meaningful [20].

At the neurophysiological level, DEPANX symptoms are considered to be related to disordered brain connectivity in large-scale networks [21–28]. In particular, three core neurocognitive networks are believed to be critically involved in DEPANX symptoms [29, 30]: the Default Mode Network (DMN), the Salience Network (SN), and the Central Executive Network (CEN). These networks play a crucial role in several cognitive processes known to be altered in both anxiety and depression, including the detection of salient stimuli (i.e., SN), top-down control (i.e., CEN), and self-referential or internally oriented mental activity (i.e., DMN) [29]. These neural systems show a high level of synchrony, particularly during the resting-state (RS) condition, which is thought to reflect the neurophysiological baseline rhythm and stable aspects of brain organization related to cognitive and affective functioning [31–35].

Despite the identification of multiple risk factors for DEPANX symptoms, relatively few studies have examined their longitudinal impact, particularly on the neural dynamics of resting-state networks (RSNs) that may be associated with changes in symptom severity over time [36, 37]. Identifying these brain-based longitudinal mechanisms in young adults is considered crucial for improving prevention and treatment strategies [37].

Given these premises, the primary aim of this study was to examine the relationship between the electrophysiological dynamics of the three core RSNs and 12-month longitudinal changes in DEPANX symptoms. Importantly, electroencephalogram (EEG) connectivity-based measures, such as EEG coherence, provide millisecond-level precision in a non-invasive and ecological manner [25], and allow the identification of distinct electrophysiological patterns that characterize large-scale networks across different frequency bands [38, 39]. Notably, RS-EEG connectivity is also considered a valid, useful, and promising biomarker for both anxiety [40, 41] and depression [42–44].

A further aim of the present research was to determine the independent contribution of RSNs to changes in DEPANX symptoms, controlling for other potential risk factors, including biological sex and familial risk for DEPANX. According with the triple-network perspective [29, 30], we hypothesized that deterioration of DEPANX symptoms would be associated with altered EEG connectivity within RSNs.

## Methods

### Participants

Recruitment took place from April 2024 to December 2025. Inclusion criteria for the present study were: age between 18 and 34 years (i.e., an age range vulnerable to the onset of both anxiety and depressive disorders [45]), both sexes, right-hand dominance (i.e., Laterality Quotient ≥ 50 [46]), and native Italian speakers. Participants were excluded if any of the following criteria were met: i) current and/or past self-reported diagnosis of psychiatric and/or neurological disorders; ii) self-reported use of psychoactive and/or illicit substances during the past two weeks; iii) familial risk for psychosis and/or bipolar disorder according to the Family History Screen [FHS; 47]. Ethical approval for this study was granted by the Ethics Review Committee of the European University of Rome (Protocol No. 12/2023), in accordance with the Declaration of Helsinki.

A total of 264 individuals were pre-screened for eligibility through an online survey distributed via web-based tools (e.g., social media). The survey included questions assessing family history of anxiety and/or depression in first-degree relatives, study inclusion/exclusion criteria, and a self-report battery. Subjects responded by opening a QR code or the link to the online survey form on their preferred device (e.g., smartphone, tablet, or PC). All participants took part anonymously and voluntarily in the research (i.e., without any form of compensation) after providing written informed consent.

Within the following two weeks, participants who reported a suspected family history of anxiety and/or depression were contacted to take part in the full experimental procedure, which included administration of the Italian translation of the FHS [47] and a baseline EEG recording (Time #1). One hundred and thirty participants (95 females; mean age: 21.51 ± 2.56 years) met the study’s inclusion/exclusion criteria and completed the baseline assessment. Of the total sample, ninety-five participants (70 females; mean age: 21.35 ± 2.65 years) completed a 12-month follow-up (Time #2), during which levels of DEPANX symptoms were assessed again. Specifically, 12 months after baseline, participants received a link to complete a new online survey. In addition to DEPANX symptoms at Time #2, participants were also asked to provide the following data: use of psychiatric medications in the past two weeks, cannabis use in the last 12 months, and details regarding the status of any psychotherapy in the last 12 months (i.e., no psychotherapy in the past year, psychotherapy started/or continued in the past year). Participant matching was performed using an alphanumeric coding system. Participants who did not complete the Time #2 survey were classified as non-completers. To assess possible attrition bias, a between-groups comparison (completers vs. non-completers) for the main socio-demographic and self-report data was performed. The two groups did not differ on most socio-demographic and clinical variables, except for educational level (see supplementary Table 1).

This research is part of a larger project examining risk factors for depression. Therefore, the sample included here partially overlaps with that presented in a previously published article by our research group [48–50]. For the present study, an a priori power analysis was conducted using G*Power 3.1 software [51] to determine the required sample size for both correlation and linear regression analyses, as needed to achieve the study aims. For the correlation analysis, the software indicated that, at a probability level of 0.05, a sample size of 82 is required to achieve acceptable statistical power (1–β = 80%) to detect a medium effect size in a two-tailed test. This sample size was also suitable for a linear regression analysis, considering an effect size of f^2^ = 0.15, with a maximum of three-tested predictors (i.e., connectivity data for DMN, SN and/or CEN) and fourteen predictors in total (see data analytic plan). Given the novelty of the study, a medium effect size was selected in accordance with recommendations for sample size estimation in human electrophysiology [52].

### Assessment procedure

The following sociodemographic and clinical data were obtained from participants who completed the survey at baseline: age, biological sex, educational level, occupation, tobacco use, lifetime or current mental and/or neurological diagnosis, use of psychiatric medications, and use of illicit substances (i.e., cannabis, cocaine or amphetamines, heroin or other opiates, and/or other unlisted illicit substances) in the past two weeks.

Levels of DEPANX symptoms were assessed using the depression and anxiety subscales of the Brief Symptom Inventory (BSI; [53]). Each subscale comprises six items measuring common manifestations of depression (i.e., *“Thoughts of ending your life”, “Feeling lonely”, “Feeling blue”, “Feeling no interest in things”, “Feeling hopeless about the future”, “Feelings of worthlessness”*) and anxiety (i.e., “*Nervousness or shakiness inside”, “Suddenly scared for no reason”*, “*Feeling fearful”,* “*Feeling tense or keyed up”, “Spells of terror or panic”,* “*Feeling so restless you couldn’t sit still”*). Responses are provided on a 5-point Likert scale ranging from 0 (‘*not at all*’) to 4 (‘*extremely*’), with higher scores indicating greater severity of DEPANX symptoms. According to previous reports e.g., [54, 55] and in line with the aim of the current research, a combined score for the depression and anxiety subscales was computed (i.e., the BSI-12; [54]). In the current study, the Italian version [56] of the subscales was used, and McDonald’s Omega was 0.90 and 0.89 for the BSI-12 total score, at Time #1 and Time #2, respectively.

Emotional dysregulation was assessed using the short-form of the Difficulties in Emotion Regulation Scale (DERS-SF; [57]), a self-report questionnaire commonly used to evaluate how individuals manage their emotions. This self-report includes 18 items rated on a 5-point Likert scale from 1 (‘*not at all true’*) to 5 (‘*completely true*’), with higher scores indicating greater difficulties in emotion regulation. The DERS-SF comprises the same six dimensions as the original scale [58]: awareness (DERS-AW), clarity (DERS-CLA), goal (DERS-GO), impulse (DERS-IMP), non-acceptance (DERS-NAP) and strategies (DERS-STR). This scale is characterized by solid psychometric properties [57, 59], comparable to the original 36-item measure. For the present study, the Italian version of the DERS-SF [59] was used, and the McDonald’s Omega ranged from 0.72 for the DERS-AW dimension to 0.94 for the DERS-IMP dimension.

ACEs were assessed using the 10-item version of the Adverse Childhood Experiences International Questionnaire (ACE-IQ-10; [60, 61]). This questionnaire comprises 10 items with dichotomous response options (i.e., 0 = “no”; 1 = “yes”), investigating five types of maltreatment (e.g., physical and emotional abuse) and five forms of family dysfunction (e.g., parental separation). Total scores range from 0 to 10, with higher values indicating a greater number of ACEs. Several studies e.g., [62, 63] indicate that the scale has suitable psychometric properties. In the present study, the Italian translation [64] of the scale was used and the McDonald’s Omega was 0.62 (item#5 was not considered as it had no variance).

Binge drinking severity was assessed using the third item (i.e., “*How often do you have six or more drinks on one occasion?*” Response options: never = 0, less than monthly = 1, monthly = 2, weekly = 3, daily or almost daily = 4) of the Alcohol Use Disorders Identification Test [65]. This item is considered a suitable tool for assessing binge drinking severity [66, 67].

Lastly, the FHS [47, 68] was used to assess the presence of mental disorders in biological first-degree relatives (i.e., parents, brothers, and sisters). In the present study, a positive response to item #9 (i.e., feeling sad, blue, or depressed for most of the time for at least two weeks) and/or item #10 (i.e., feeling quite tired, having less energy, or not caring about usual activities for at least two weeks) was used to identify individuals with a familial risk for depression (FHS-DEP). Furthermore, positive responses to items #13–14 (panic attacks), #15 (generalized anxiety disorder), #16 (agoraphobia), #17 (specific phobias), and/or #18 (social anxiety disorder) were used to identify individuals with a familial risk for anxiety disorders (FHS-ANX).

### EEG data acquisition and analysis

After completing the FHS, participants underwent a RS-EEG recording in the EEG laboratory of the European University of Rome. They were instructed to avoid caffeinated and alcoholic beverages for at least four hours prior to the recording (participants were again asked about the absence of substance use in the preceding two weeks). Each EEG session lasted at least five minutes and was conducted in a quiet room while participants remained comfortably seated with their eyes closed. A 64-channel amplifier (BrainAmp, BrainProducts GmbH, Gilching, Germany) with passive electrodes located according to the international 10–10 system was used (the reference electrode was positioned at FCz). Impedances were kept below 20 kΩ throughout the recordings, and data were sampled online at 500 Hz. A qualitative visual inspection of the raw EEG data was performed to identify any signs of abnormal neurophysiological activity or drowsiness before EEG preprocessing and analysis. Offline EEG processing was carried out using the EEGLAB toolbox for MATLAB (version 2022.1; [69]). The EEG data were first downsampled to 256 Hz, then band-pass filtered (1–40 Hz), and subsequently re-referenced to the average reference. Following an initial visual inspection, artifacts were removed using Independent Component Analysis (ICA) implemented with the Infomax algorithm. In cases of bad channels, three-dimensional spherical spline interpolation [70] was also applied.

Artifact-cleaned EEG data were then imported and analyzed using the Exact Low-Resolution Electromagnetic Tomography software [eLORETA; 71, a recognized digital tool for assessing electrocortical activity [72–74] and investigating brain networks connectivity [75]. Although the eLORETA has low spatial resolution, it reliably reconstructs electrophysiological activity via a three-dimensional (3D), linear, distributed non-inverse norm solution [76].

To evaluate EEG connectivity within each RSNs (i.e., intra-network analysis), Regions of Interest (ROIs) for the DMN, CEN, and SN were defined (supplementary Table 2) according to previous eLORETA studies [77–79]. Using the Montréal Neurological Institute coordinates, ROIs were generated with the ‘ROI-maker#1’ option in eLORETA. In line with prior studies e.g., [80–82], each ROI (i.e., 7 for the SN, 6 for the CEN and 6 for the DMN) was defined using the ‘single nearest voxel’ approach (i.e., each ROI consisted of the voxel closest to each RSN node).

Following the guidelines of Miljevic et al. [83], artifact-free EEG data were segmented into 4-s non-overlapping epochs before connectivity analysis using the eLORETA software. Only EEG traces with at least 50 epochs (≥ 200 s) per participant were considered suitable and included in the analysis. In the current sample, the mean number of epochs analyzed was 66.96 ± 6.51 (range: 51–77). After segmentation, functional connectivity within RSN nodes was assessed using the lagged phase synchronization (LPS) algorithm [71]. This algorithm is a widely used metric for EEG connectivity e.g., [84, 85], as it eliminates instantaneous zero-lag contributions and reduces the influence of non-physiological artifacts. By employing the normalized Fourier transform, LPS quantifies the synchrony of EEG signals within specific frequency bands, resulting in values from 0 (no connection between two nodes) to 1 (maximal connection between two nodes). In this study, LPS was analyzed in the delta (1–4 Hz), theta (4.5–7.5 Hz), alpha (8–13 Hz), and beta (13.5–30 Hz) frequency ranges.

### Data analytic plan

To assess changes in DEPANX symptoms between Time #1 and Time #2, residual change score (RCS) analysis [86] was performed. This index is derived by computing the residuals from a linear regression in which DEPANX symptoms at Time #1 are used to predict DEPANX symptoms at Time #2 [87]. Thus, these scores represent the proportion of variance in symptom change not explained by previous levels of symptomatology (i.e., Time #1 scores). In other words, these scores reflect whether the observed degree of change for a particular participant was greater or less than the change expected linearly from his or her baseline score [87]. A positive RCS reflects deterioration (e.g., Time #2 is greater than time #1, or a lower-than-expected reduction in symptoms), and a negative change score indicates improvement (e.g. time #2 score is lower than time #1, or a higher-than-expected reduction in symptoms) [88, 89].

This approach is a widely used method for assessing change beyond simple differences between time points, helping to reduce statistical issues, such as the problem of regression to the mean (i.e., extreme scorers are likely to show more change when assessed a second time than those with more moderate initial scores) [90, 91]. In light of the above, these values (i.e., RCS-DEPANX) were included in all analyses.

To investigate the association between intra-network functional connectivity and RCS-DEPANX, we conducted a primary analysis using the regression analysis option in the eLORETA software. This analysis formally corresponds to a series of correlation analyses between each ROI and an external variable (i.e., RCS-DEPANX was correlated with the strength of the connections between each ROI, in each frequency band, within each defined RSN). This analysis was performed using the statistical non-parametric mapping method [92] with 5000 randomizations to determine the critical probability thresholds for the *r*-values and to calculate the associated statistically adjusted *p*-values (*p* = 0.05 and *p* = 0.01). This procedure is considered a valid strategy for addressing the multiple testing problem, as it corrects for multiple comparisons among all connections in each frequency band without requiring Gaussianity [for technical details see 84].

As a sensitivity analysis, partial correlation (*r*_p_) analyses were performed between significant EEG connectivity data identified by eLORETA and BSI-DEP, BSI-ANX, and DEPANX scores at Time #2, adjusting separately for the corresponding baseline values. As an additional sensitivity analysis, an independent t-test was performed comparing participants with low versus high severity in pre–post DEPANX score differences (Δ = post − pre), using tertile-based cut-offs (33rd and 66th percentiles) of the sample distribution.

As a secondary analysis, a Pearson correlation analysis was also performed to investigate the association between self-report data (i.e., ACE total score, DERS dimensions, and AUDIT#3) and RCS-DEPANX. Lastly, to investigate the unique contribution of intra-network functional connectivity data to the RCS-DEPANX, a linear regression analysis was conducted using only the significantly interconnected ROIs and correlated variables identified in the primary and secondary analyses as predictors, with RCS-DEPANX as the dependent variable. Potential confounding variables (i.e., familial risk for DEP and ANX, biological sex, age, occupation, educational level, Time #1 tobacco use, cannabis use in the last 12 months, Time #2 psychotherapy status) were also included in the model. Predictors were entered simultaneously into the regression. The normality of self-report data was assessed using skewness and kurtosis [93]. Non-normally distributed variables were subjected to square root transformation. Multiple regression assumptions were tested in accordance with Williams and colleagues [94]. Multicollinearity was assessed by calculating the tolerance value and the Variance Inflation Factor (VIF) for each variable. Influential data points were identified using Cook’s distances. Results were reported as standardized beta (*β*) coefficients and corresponding 95% confidence intervals (CI). Pearson correlation and linear regression analyses were conducted using SPSS version 20 (IBM, Armonk, NY, USA).

## Results

The descriptive statistics for the current sample are shown in Table 1. At Time #2, no participants reported using psychiatric medications or illegal substances in the past two weeks, while 41 (43.2%) individuals either initiated or continued psychotherapy over the past year. DEPANX scores at the two time points and their relationship with RCS-DEPANX are presented graphically in supplementary Fig. 1. No significant differences were observed in DEPANX scores between Time #1 and Time #2 (t_94_ = 1.629, *p* = 0.107).

**Table 1:** Descriptive statistics of the sample (N = 95)

Age —M ± SD	21.35 ± 2.65
Females—N (%)	70 (73.7)
Students—N (%)	87 (91.6)
Bachelor’s Degree or higher—N (%)	20 (21.1)
Tobacco use—N (%)	35 (36.8)
Cannabis use in the last 12 months—N (%)	27 (28.4)
Psychotherapy in last 12 months—N (%)	41 (43.2)
FHS-DEP—N (%)	19 (20)
FHS-ANX—N (%)	13 (13.7)
FHS-DEPANX—N (%)	30 (31.6)
AUDIT #3—M ± SD	0.64 ± 0.89
ACE-IQ-10—M ± SD	1.56 ± 1.57
DERS-AW—M ± SD	6.20 ± 2.57
DERS-CLA—M ± SD	6.51 ± 2.81
DERS-GO—M ± SD	9.33 ± 3.33
DERS-IMP—M ± SD	5.78 ± 3.28
DERS-NAP—M ± SD	6.28 ± 3.11
DERS-STR—M ± SD	6.45 ± 3.10
BSI-DEPANX Time# 1—M ± SD	2.11 ± 1.51
BSI-DEPANX Time# 2—M ± SD	1.89 ± 1.42

*M* mean ; *SD*   standard deviation

*FHS-DEP*   familial risk of depression according to the family history screen, *FHS-ANX*   familial risk of anxiety according to the family history screen anxiety, *FHS-DEPANX*  familial risk of depression and anxiety according to the family history screen anxiety, *AUDIT*#3   item#3 (i.e., binge drinking) of the Alcohol Use Disorders Identification Test, *ACE-IQ*-10   10-item version of the Adverse Childhood Experiences International Questionnaire, *DERS*   Difficulties in Emotion Regulation Scale, *DERS-AW*   awareness, *DERS-CLA*  clarity, *DERS*-*GO*   goal, *DERS-IMP*   impulse, *DERS-NAP*  non-acceptance *DERS-STR*   strategies, *BSI*  brief symptom inventory *DEP*  depressive symptoms, *ANX*   anxiety symptoms

### Primary and sensitivity analyses

**Fig. 1:**
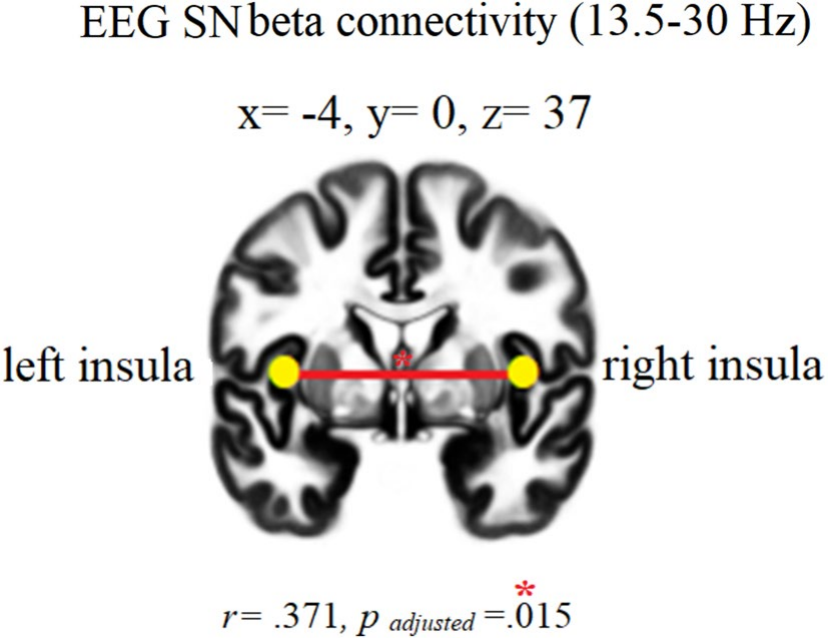
Significant correlation between salience network (SN) connectivity and residualchange score of depression and anxiety (RCS-DEPANX) in the beta frequency band. Red lines indicate a positive correlation between RCS-DEPANX and the strength of connections between two interconnected nodes. Blue lines (not present) would indicate a negative correlation

Regarding the association between RCS-DEPANX and RSNs, a significant positive correlation was found in the SN. In this eLORETA analysis, the thresholds for significance (corrected for multiple comparisons) were *r* =  ± 0.381 (corresponding to *p*_*adjusted*_ = 0.01) and *r* =  ± 0.341 (corresponding to* p*_*adjusted*_ = 0.05). A significant association between RCS-DEPANX and EEG connectivity data was observed in the beta frequency band (Fig. 1) between the right and left insula (*r* = 0.371; *p*_*adjusted*_ = 0.015). This connectivity pattern was also positively associated with BSI-DEP (*r*_p_ = 0.324, *p* = 0.001), BSI-ANX (*r*_p_ = 0.315, *p* = 0.002), and DEPANX (*r*_p_ = 0.348, *p* = 0.001) scores at Time #2, after controlling for the corresponding baseline values.^1^ Lastly, the same connectivity pattern also differed significantly between participants with low (N = 34, 27 females) and high (N = 33, 26 females) levels of change in DEPANX symptom severity (t_54,17_ = -2.29, *p* = 0.026).

### Secondary analyses

Analyses of self-report data showed that binge drinking severity (*r* = 0.279, *p* = 0.006) and DERS-IMP (*r* = 0.211, *p* = 0.041) at Time #1 were positively associated with RCS-DEPANX. No other significant correlations were detected (see supplementary Table 3).

The results of the linear regression analysis are shown in Table 2. All linear regression assumptions were satisfied, including heteroscedasticity (i.e., Breusch-Pagan test F_12;82_ = 1.285; *p* = 0.243). Durbin-Watson test was = 1.836. The tolerance and VIF statistics indicated no relevant multicollinearity (i.e., tolerance values > 0.10 and VIF < 5), and Cook’s distances were acceptable (i.e., maximum value = 0.121). The models explained 15% of the variability of the RCS-DEPANX. When controlling for the presence of other variables (i.e., familial risk for DEP and ANX, biological sex, age, occupation, educational level, Time #1 tobacco use, cannabis use in the last 12 months, Time #2 psychotherapy status), beta connectivity values remained positively and independently associated with RCS-DEPANX (*β* = 0.328; *p* = 0.002); thus, increased SN beta connectivity was associated with greater deterioration of DEPANX symptoms at Time #2 when controlling for potential confounding variables. No other significant associations were found. Unadjusted associations between study variables and RCS-DEPANX, as well as associations among independent variables, are presented in supplementary Table 4 and supplementary Table 5, respectively.

**Table 2:** Linear regression analysis (N = 95)

Dependent variable	Adjusted R^2^	*F*_12:82_	Independent variable	*β*	95% CI	*p*
RCS-DEPANX	0.152	2.403				**0.010**
			FHS-DEP	−0.080	[−0.581; .264]	0.458
			FHS-ANX	0.008	[−0.401; .431]	0.942
			Sex	−0.005	[−0.476; .455]	0.964
			Age	0.020	[−0.089; .104]	0.880
			Student	−0.015	[−0.842; .736]	0.894
			Bachelor’s degree or higher	−0.096	[−0.802; .336]	0.417
			Psychotherapy in last 12 months	−0.037	[−0.467; .320]	0.710
			Tobacco use	0.025	[−0.417; .520]	0.827
			Cannabis use in the last 12 months	0.180	[−0.102; .893]	0.118
			AUDIT#3	0.204	[−0.026; .484]	0.078
			DERS-IMP	0.133	[−0.023; .104]	0.208
			Bilateral insula beta connectivity	0.328	[0.124; .529]	**0.002**

In bold significant values; connectivity data are expressed as standardized values; Psychotherapy and Cannabis data use were assessed at Time #2

*RCS-DEPANX*   residual change score of depression and anxiety symptoms, *FHS-DEP*   familial risk of depression according to the family history screen, *FHS-ANX* = familial risk of anxiety according to the family history screen anxiety, *AUDIT*#3  item#3 (i.e., binge drinking) of the Alcohol Use Disorders Identification Test, *DERS-IMP*   impulse dimension of the Difficulties in Emotion Regulation Scale

## Discussion

According to the triple-network model of DEPANX symptomatology [29, 30], we examined whether intra-network EEG connectivity within the DMN, SN, and CEN was associated with longitudinal changes in symptoms over a 12-month period. In this study, we found that baseline SN connectivity—particularly bilateral insula beta coherence—was associated with DEPANX longitudinal symptom changes over 12 months, whereas no comparable longitudinal associations were observed for DMN or CEN connectivity. At the bivariate level, and consistent with previous reports [16, 95–100], our results also showed that substance misuse patterns (such as binge drinking severity and cannabis use) and impulse control difficulties were associated with changes in DEPANX symptoms over 12 months. However, at the multivariate level, our findings indicated that only SN beta connectivity remained independently associated with DEPANX longitudinal changes, suggesting a specific role for the bilateral insula in predicting deterioration over a 12-month period.

The SN is a crucial brain system involved in cognitive and affective information processing, supporting the detection and filtering of salient stimuli, attentional modulation, and dynamic switching between other neural networks [101, 102]. Although it is composed of several nodes, including the dorsal anterior cingulate cortex, prefrontal cortex, and supramarginal gyrus [103], the insular cortex is considered the key hub of this network [101], “*acting as a gatekeeper of executive control*” [102].

This area modulates autonomic responses and generates signals sent to other SN nodes, selectively amplifying salient stimuli that require further cortical processing [102]. Aberrantly increased bilateral insula co-activation and connectivity have been linked to the anticipation of negative stimuli in anxious individuals [104]. Similarly, increased bilateral insula activity has been observed in patients with depression during the processing of negative emotional faces [105]. Notably, at the electrophysiological level, increased RS beta coherence has been interpreted as a marker of neural hyper-excitability and hyper-synchronization [106, 107]. Furthermore, enhanced event-related beta synchronization in the bilateral insula has been proposed to play a key role in the integrative processing of task-relevant versus irrelevant stimuli [108].

According to cognitive models of anxiety and depression, increased attention to disorder-relevant negative information (i.e., negative attentional biases) characterizes these conditions and is thought to play a major role in their development and maintenance [109]. Thus, given this background, the observed association between increased baseline RS beta connectivity and 12-month changes in DEPANX symptoms may reflect a longitudinal change of misattributing salience to non-salient events. This is consistent with previous research [29, 102] linking increased SN activity and connectivity in anxiety and depression to the abnormal attribution of importance to negative stimuli, which can lead to the generalization of stress responses in non-threatening situations.

Intriguingly, and consistent with our interpretation, several neuroimaging studies have reported reduced activation in key SN regions, including the insula, following psychological therapies in patients with anxiety and depression, in line with neural models of improved emotion regulation and attentional control [110]. Similarly, a recent investigation in depression has shown that clinical response to repetitive transcranial magnetic stimulation is associated with reduced functional connectivity within the SN after treatment [111]. Although our interpretation is compelling, it remains largely speculative and should be treated with caution, as the present study did not assess attentional bias at baseline or follow-up.

In the present study, we did not find significant associations between baseline DMN/CEN connectivity and 12-month changes in DEPANX symptoms. Several possible explanations may account for these null findings. First, our sample consisted of non-clinical young adults. Thus, it is possible that, in patients with depression and/or anxiety, different connectivity patterns may be associated with DEPANX longitudinal changes. Second, a 12-month period may be insufficient to detect connectivity patterns within these networks associated with changes in DEPANX symptoms over time. Moreover, DEPANX longitudinal changes may be more sensitively identified using task-based paradigms (e.g., repetitive negative thinking or Go/NoGo tasks) rather than RS assessments at baseline. Lastly, it is also possible that, with regard to the CEN and DMN, anxiety and depressive symptoms follow independent longitudinal changes. This latter hypothesis is consistent with EEG coherence data suggesting that anxiety [112–114] and depression [38, 85, 115] may be characterized by distinct connectivity configurations.

### Strengths and limitations

Beyond the limitations already highlighted (i.e., the short follow-up period, the absence of baseline task-related EEG recordings, the lack of attentional bias assessment, and the absence of a clinical sample), additional considerations merit attention. First, although individuals with self-reported past or current psychiatric diagnoses were not included in the present research, no comprehensive structured clinical interview was conducted. DEPANX symptoms were also assessed using a self-report measure, which may be subject to various biases, including social desirability. Third, the present sample consisted predominantly of university students and female participants, which may limit the generalizability of our findings. Fourth, the assessment of potential traumatic events, such as experiences of bereavement, in the preceding 12 months was not conducted. Finally, although eLORETA is a suitable software for assessing large-scale brain networks [75] and for localizing paralimbic and limbic regions [116], it precludes the detection of subcortical structures (e.g., the thalamus) that are components of several RSNs [117] and may be involved in both anxiety [118] and depression [119] symptom change. Given these limitations, future studies should consider a longer follow-up, a clinical sample, combine multimodal brain imaging techniques, and investigate

neural synchronization using task-based paradigms as well. Despite current limitations, by examining the relationship between RSN dynamics and DEPANX longitudinal symptom changes over a 12-month period, our data provide further evidence in a research area that remains relatively understudied.

## Conclusions

Overall, our results showed that baseline SN connectivity was independently associated with longitudinal changes in DEPANX, suggesting a specific role for the bilateral insula in predicting deterioration over a 12-month period. If replicated in future studies, this finding may represent a potential early indicator of symptom progression. Identifying these brain-based longitudinal mechanisms in young adults is crucial for improving prevention and treatment strategies [37]. From a therapeutic perspective, our data suggest that interventions targeting SN connectivity, especially the bilateral insula, may help mitigate DEPANX symptom deterioration in young adults.

## Supplementary Information

Below is the link to the electronic supplementary material.

Supplementary file1 (PDF 655 KB)

## Data Availability

Aggregated data are available from the first author upon request.

## Footnotes

1 SN connectivity data, DEPANX, and BSI-DEP scores were square-root transformed before analysis.

## References

[1] Jang W, Cho H, Kim S et al (2026) Global burden of anxiety, depression, and self-harm (1990–2021) and the impact of the COVID-19 pandemic. J Affect Disord 392:120201. 10.1016/j.jad.2025.120201 40915503 10.1016/j.jad.2025.120201

[2] Liu W, Zhang Y, Chen J, Li X, Huang Y, Zhao F, Chen F, Qu P, Li Y (2025) Global burden and trends of major mental disorders in individuals under 24 years of age from 1990 to 2021, with projections to 2050: insights from the Global Burden of Disease Study 2021. Front Public Health 13:1635801. 10.3389/fpubh.2025.1635801 41036122 10.3389/fpubh.2025.1635801 PMC12481897

[3] Zhang Z, Chen X, Wu S et al (2026) Global, regional and national burden of anxiety and depression disorders from 1990 to 2021, and forecasts up to 2040. J Affect Disord 393:120299. 10.1016/j.jad.2025.120299 40935255 10.1016/j.jad.2025.120299

[4] Li W, Zhao Z, Chen D, Peng Y, Lu Z (2022) Prevalence and associated factors of depression and anxiety symptoms among college students: a systematic review and meta-analysis. J Child Psychol Psychiatry 63:1222–1230. 10.1111/jcpp.13606 35297041 10.1111/jcpp.13606

[5] Saha S, Lim CCW, Cannon DL, Burton L, Bremner M, Cosgrove P, Huo Y, J JM (2021) Co-morbidity between mood and anxiety disorders: a systematic review and meta-analysis. Depress Anxiety 38:286–306. 10.1002/da.23113 33225514 10.1002/da.23113 PMC7984258

[6] Rasing SPA, Creemers DHM, Janssens J, Scholte RHJ (2017) Depression and anxiety prevention based on cognitive behavioral therapy for at-risk adolescents: a meta-analytic review. Front Psychol 8:1066. 10.3389/fpsyg.2017.01066 28701980 10.3389/fpsyg.2017.01066 PMC5487592

[7] Marx W, Penninx B, Solmi M, Furukawa TA, Firth J, Carvalho AF, Berk M (2023) Major depressive disorder. Nat Rev Dis Primers 9:44. 10.1038/s41572-023-00454-1 37620370 10.1038/s41572-023-00454-1

[8] Nugent NR, Tyrka AR, Carpenter LL, Price LH (2011) Gene-environment interactions: early life stress and risk for depressive and anxiety disorders. Psychopharmacology 214:175–196. 10.1007/s00213-010-2151-x 21225419 10.1007/s00213-010-2151-x PMC3615637

[9] Gotlib IH, Joormann J, Foland-Ross LC (2014) Understanding familial risk for depression: a 25-year perspective. Perspect Psychol Sci 9:94–108. 10.1177/1745691613513469 26173248 10.1177/1745691613513469 PMC11877285

[10] Dachew B, Ayano G, Duko B, Lawrence B, Betts K, Alati R (2023) Paternal depression and risk of depression among offspring: a systematic review and meta-analysis. JAMA Netw Open 6:e2329159. 10.1001/jamanetworkopen.2023.29159 37585203 10.1001/jamanetworkopen.2023.29159 PMC10433087

[11] Lawrence PJ, Murayama K, Creswell C (2019) Systematic review and meta-analysis: anxiety and depressive disorders in offspring of parents with anxiety disorders. J Am Acad Child Adolesc Psychiatry 58:46–60. 10.1016/j.jaac.2018.07.898 30577938 10.1016/j.jaac.2018.07.898

[12] Yu P, Jiang Z, Zheng C, Zeng P, Huang L, Jin Y, Wang K (2023) Variety aces and risk of developing anxiety, depression, or anxiety-depression co-morbidity: the 2006-2022 UK biobank data. Front Psychiatry 14:1233981. 10.3389/fpsyt.2023.1233981 38234367 10.3389/fpsyt.2023.1233981 PMC10793109

[13] Zhu S, Cheng S, Liu W et al (2025) Gender differences in the associations of adverse childhood experiences with depression and anxiety: a systematic review and meta-analysis. J Affect Disord 378:47–57. 10.1016/j.jad.2025.02.074 39988140 10.1016/j.jad.2025.02.074

[14] Kundakovic M, Rocks D (2022) Sex hormone fluctuation and increased female risk for depression and anxiety disorders: from clinical evidence to molecular mechanisms. Front Neuroendocrinol 66:101010. 10.1016/j.yfrne.2022.101010 35716803 10.1016/j.yfrne.2022.101010 PMC9715398

[15] Schafer JO, Naumann E, Holmes EA, Tuschen-Caffier B, Samson AC (2017) Emotion regulation strategies in depressive and anxiety symptoms in youth: a meta-analytic review. J Youth Adolesc 46:261–276. 10.1007/s10964-016-0585-0 27734198 10.1007/s10964-016-0585-0

[16] Garey L, Olofsson H, Garza T, Rogers AH, Kauffman BY, Zvolensky MJ (2020) Directional effects of anxiety and depressive disorders with substance use: a review of recent prospective research. Curr Addict Rep 7:344–355. 10.1007/s40429-020-00321-z

[17] Lannoy S, Duka T, Carbia C et al (2021) Emotional processes in binge drinking: a systematic review and perspective. Clin Psychol Rev 84:101971. 10.1016/j.cpr.2021.101971 33497920 10.1016/j.cpr.2021.101971 PMC8275688

[18] Sham PC, Sterne A, Purcell S et al (2000) Genesis: creating a composite index of the vulnerability to anxiety and depression in a community-based sample of siblings. Twin Res 3:316–322. 10.1375/136905200320565292 11463153 10.1375/136905200320565292

[19] Hong RY, Cheung MWL (2015) The structure of cognitive vulnerabilities to depression and anxiety: evidence for a common core etiologic process based on a meta-analytic review. Clin Psychol Sci 3:892–912. 10.1177/2167702614553789

[20] Moller HJ, Bandelow B, Volz HP, Barnikol UB, Seifritz E, Kasper S (2016) The relevance of “mixed anxiety and depression” as a diagnostic category in clinical practice. Eur Arch Psychiatry Clin Neurosci 266:725–736. 10.1007/s00406-016-0684-7 27002521 10.1007/s00406-016-0684-7 PMC5097109

[21] Sylvester CM, Corbetta M, Raichle ME, Rodebaugh TL, Schlaggar BL, Sheline YI, Zorumski CF, Lenze EJ (2012) Functional network dysfunction in anxiety and anxiety disorders. Trends Neurosci 35:527–535. 10.1016/j.tins.2012.04.012 22658924 10.1016/j.tins.2012.04.012 PMC3432139

[22] Northoff G (2020) Anxiety disorders and the brain’s resting state networks: from altered spatiotemporal synchronization to psychopathological symptoms. Adv Exp Med Biol 1191:71–90. 10.1007/978-981-32-9705-0_5 32002923 10.1007/978-981-32-9705-0_5

[23] Xu J, Van Dam NT, Feng C, Luo Y, Ai H, Gu R, Xu P (2019) Anxious brain networks: a coordinate-based activation likelihood estimation meta-analysis of resting-state functional connectivity studies in anxiety. Neurosci Biobehav Rev 96:21–30. 10.1016/j.neubiorev.2018.11.005 30452934 10.1016/j.neubiorev.2018.11.005

[24] Li BJ, Friston K, Mody M, Wang HN, Lu HB, Hu DW (2018) A brain network model for depression: from symptom understanding to disease intervention. CNS Neurosci Ther 24:1004–1019. 10.1111/cns.12998 29931740 10.1111/cns.12998 PMC6490158

[25] Miljevic A, Bailey NW, Murphy OW, Perera MPN, Fitzgerald PB (2023) Alterations in EEG functional connectivity in individuals with depression: a systematic review. J Affect Disord 328:287–302. 10.1016/j.jad.2023.01.126 36801418 10.1016/j.jad.2023.01.126

[26] Helm K, Viol K, Weiger TM, Tass PA, Grefkes C, Del Monte D, Schiepek G (2018) Neuronal connectivity in major depressive disorder: a systematic review. Neuropsychiatr Dis Treat 14:2715–2737. 10.2147/NDT.S170989 30425491 10.2147/NDT.S170989 PMC6200438

[27] Kaiser RH, Andrews-Hanna JR, Wager TD, Pizzagalli DA (2015) Large-scale network dysfunction in major depressive disorder: a meta-analysis of resting-state functional connectivity. JAMA Psychiat 72:603–611. 10.1001/jamapsychiatry.2015.0071 10.1001/jamapsychiatry.2015.0071 PMC4456260 25785575

[28] Zhang Z, Zhang Y, Wang H et al (2025) Resting-state network alterations in depression: a comprehensive meta-analysis of functional connectivity. Psychol Med 55:e63. 10.1017/S0033291725000303 40008424 10.1017/S0033291725000303 PMC12080655

[29] Menon B (2019) Towards a new model of understanding - the triple network, psychopathology and the structure of the mind. Med Hypotheses 133:109385. 10.1016/j.mehy.2019.109385 31494485 10.1016/j.mehy.2019.109385

[30] Menon V (2011) Large-scale brain networks and psychopathology: a unifying triple network model. Trends Cogn Sci 15:483–506. 10.1016/j.tics.2011.08.003 21908230 10.1016/j.tics.2011.08.003

[31] Anderson AJ, Perone S (2018) Developmental change in the resting state electroencephalogram: insights into cognition and the brain. Brain Cogn 126:40–52. 10.1016/j.bandc.2018.08.001 30144749 10.1016/j.bandc.2018.08.001

[32] Deco G, Jirsa VK, McIntosh AR (2011) Emerging concepts for the dynamical organization of resting-state activity in the brain. Nat Rev Neurosci 12:43–56. 10.1038/nrn2961 21170073 10.1038/nrn2961

[33] Blanken TF, Bathelt J, Deserno MK, Voge L, Borsboom D, Douw L (2021) Connecting brain and behavior in clinical neuroscience: a network approach. Neurosci Biobehav Rev 130:81–90. 10.1016/j.neubiorev.2021.07.027 34324918 10.1016/j.neubiorev.2021.07.027

[34] Smith SM, Vidaurre D, Beckmann CF et al (2013) Functional connectomics from resting-state fMRI. Trends Cogn Sci 17:666–682. 10.1016/j.tics.2013.09.016 24238796 10.1016/j.tics.2013.09.016 PMC4004765

[35] Shen X, Finn ES, Scheinost D, Rosenberg MD, Chun MM, Papademetris X, Constable RT (2017) Using connectome-based predictive modeling to predict individual behavior from brain connectivity. Nat Protoc 12:506–518. 10.1038/nprot.2016.178 28182017 10.1038/nprot.2016.178 PMC5526681

[36] Macedo MA, Sato JR, Bressan RA, Pan PM (2022) Adolescent depression and resting-state fMRI brain networks: a scoping review of longitudinal studies. Braz J Psychiatry 44:420–433. 10.47626/1516-4446-2021-2032 35896034 10.47626/1516-4446-2021-2032 PMC9375668

[37] Morfini F, Kucyi A, Zhang J et al (2025) Brain functional connectivity predicts depression and anxiety during childhood and adolescence: A connectome-based predictive modeling approach. Imaging Neurosci (Camb). 10.1162/IMAG.a.145 40959708 10.1162/IMAG.a.145 PMC12434380

[38] Whitton AE, Deccy S, Ironside ML, Kumar P, Beltzer M, Pizzagalli DA (2018) Electroencephalography source functional connectivity reveals abnormal high-frequency communication among large-scale functional networks in depression. Biol Psychiatry Cogn Neurosci Neuroimaging 3:50–58. 10.1016/j.bpsc.2017.07.001 29397079 10.1016/j.bpsc.2017.07.001 PMC5801763

[39] Mantini D, Perrucci MG, Del Gratta C, Romani GL, Corbetta M (2007) Electrophysiological signatures of resting state networks in the human brain. Proc Natl Acad Sci U S A 104:13170–13175. 10.1073/pnas.0700668104 17670949 10.1073/pnas.0700668104 PMC1941820

[40] Schoenberg PLA (2020) Linear and nonlinear EEG-based functional networks in anxiety disorders. Adv Exp Med Biol 1191:35–59. 10.1007/978-981-32-9705-0_3 32002921 10.1007/978-981-32-9705-0_3

[41] Trambaiolli LR, Biazoli CE (2020) Resting-state global eeg connectivity predicts depression and anxiety severity. Annu Int Conf IEEE Eng Med Biol Soc 2020:3707–3710. 10.1109/EMBC44109.2020.9176161 33018806 10.1109/EMBC44109.2020.9176161

[42] Greco C, Matarazzo O, Cordasco G, Vinciarelli A, Callejas Z, Esposito A-B (2021) Discriminative power of EEG-based biomarkers in major depressive disorder: a systematic review. IEEE Access 9:112850–112870. 10.1109/ACCESS.2021.3103047

[43] Wu CT, Huang HC, Huang S et al (2021) Resting-state eeg signal for major depressive disorder detection: A systematic validation on a large and diverse dataset. Biosensors. 10.3390/bios11120499 34940256 10.3390/bios11120499 PMC8699348

[44] Simmatis L, Russo EE, Geraci J, Harmsen IE, Samuel N (2023) Technical and clinical considerations for electroencephalography-based biomarkers for major depressive disorder. npj Ment Health Res 2:18. 10.1038/s44184-023-00038-7 38609518 10.1038/s44184-023-00038-7 PMC10955915

[45] Solmi M, Radua J, Olivola M et al (2022) Age at onset of mental disorders worldwide: large-scale meta-analysis of 192 epidemiological studies. Mol Psychiatry 27:281–295. 10.1038/s41380-021-01161-7 34079068 10.1038/s41380-021-01161-7 PMC8960395

[46] Oldfield RC (1971) The assessment and analysis of handedness: the Edinburgh inventory. Neuropsychologia 9:97–113. 10.1016/0028-3932(71)90067-4 5146491 10.1016/0028-3932(71)90067-4

[47] Weissman MM, Wickramaratne P, Adams P, Wolk S, Verdeli H, Olfson M (2000) Brief screening for family psychiatric history: The family history screen. Arch Gen Psychiatry 57:675–682. 10.1001/archpsyc.57.7.675 10891038 10.1001/archpsyc.57.7.675

[48] Imperatori C, De Rossi E, Allegrini G et al (2025) Brooding rumination as a mediator of the association between default mode and central executive network connectivity and general psychopathology: A resting state eeg study. J Affect Disord. 10.1016/j.jad.2025.120535 41284532 10.1016/j.jad.2025.120535

[49] Imperatori C, Messerotti Benvenuti S, Ardito RB et al (2026) Alpha connectivity in the frontoparietal network is increased in individuals with familial risk for depression: A resting-state eeg study towards a potential endophenotype. Behav Res Ther 202:105041. 10.1016/j.brat.2026.105041 42066390 10.1016/j.brat.2026.105041

[50] Allegrini G, De Rossi E, Bersani FS, Carbone GA, Ardito RB, Adenzato M, Farina B, Imperatori C (2026) Increased salience network connectivity in college students who engage in binge drinking: A resting state eeg study. Drug Alcohol Depend 282:113098. 10.1016/j.drugalcdep.2026.113098 41747409 10.1016/j.drugalcdep.2026.113098

[51] Faul F, Erdfelder E, Buchner A, Lang AG (2009) Statistical power analyses using g*power 3.1: Tests for correlation and regression analyses. Behav Res Methods 41:1149–1160. 10.3758/BRM.41.4.1149 19897823 10.3758/BRM.41.4.1149

[52] Larson MJ, Carbine KA (2017) Sample size calculations in human electrophysiology (eeg and erp) studies: A systematic review and recommendations for increased rigor. Int J Psychophysiol 111:33–41. 10.1016/j.ijpsycho.2016.06.015 27373837 10.1016/j.ijpsycho.2016.06.015

[53] Derogatis LR, Melisaratos N (1983) The brief symptom inventory: An introductory report. Psychol Med 13:595–605 6622612

[54] Quintana GR, Ponce FP, Escudero-Pasten JI et al (2024) Cross-cultural validation and measurement invariance of anxiety and depression symptoms: A study of the brief symptom inventory (bsi) in 42 countries. J Affect Disord 350:991–1006. 10.1016/j.jad.2024.01.127 38244805 10.1016/j.jad.2024.01.127

[55] Allen GE, Heppner PP (2011) Religiosity, coping, and psychological well-being among latter-day saint polynesians in the us. Asian Am J Psychol 2:13–24. 10.1037/a0023266

[56] Adawi M, Zerbetto R, Re TS, Bisharat B, Mahamid M, Amital H, Del Puente G, Bragazzi NL (2019) Psychometric properties of the brief symptom inventory in nomophobic subjects: Insights from preliminary confirmatory factor, exploratory factor, and clustering analyses in a sample of healthy italian volunteers. Psychol Res Behav Manag 12:145–154. 10.2147/PRBM.S173282 30881158 10.2147/PRBM.S173282 PMC6419603

[57] Kaufman EA, Xia M, Fosco G, Yaptangco M, Skidmore CR, Crowell SE (2016) The difficulties in emotion regulation scale short form (ders-sf): Validation and replication in adolescent and adult samples. J Psychopathol Behav Assess 38:443–455 41522882 10.1007/s10862-015-9529-3 PMC12788809

[58] Gratz KL, Roemer L (2004) Multidimensional assessment of emotion regulation and dysregulation: development, factor structure, and initial validation of the difficulties in emotion regulation scale. J Psychopathol Behav Assess 26:41–54. 10.1023/B:JOBA.0000007455.08539.94

[59] Rossi AA, Panzeri A, Mannarini S (2023) The Italian version of the difficulties in emotion regulation scale–short form (it-ders-sf): a two-step validation study. J Psychopathol Behav Assess 45:572–590. 10.1007/s10862-022-10006-8

[60] Felitti VJ, Anda RF, Nordenberg D, Williamson DF, Spitz AM, Edwards V, Koss MP, Marks JS (1998) Relationship of childhood abuse and household dysfunction to many of the leading causes of death in adults. The adverse childhood experiences (ACE) study. Am J Prev Med 14:245–258. 10.1016/s0749-3797(98)00017-8 9635069 10.1016/s0749-3797(98)00017-8

[61] Wingenfeld K, Schafer I, Terfehr K, Grabski H, Driessen M, Grabe H, Lowe B, Spitzer C (2011) [The reliable, valid and economic assessment of early traumatization: first psychometric characteristics of the German version of the adverse childhood experiences questionnaire (ACE)]. Psychother Psychosom Med Psychol 61:e10-14. 10.1055/s-0030-1263161 20878600 10.1055/s-0030-1263161

[62] Kovacs-Toth B, Olah B, Kuritarne Szabo I, Fekete Z (2023) Psychometric properties of the adverse childhood experiences questionnaire 10 item version (ace-10) among Hungarian adolescents. Front Psychol 14:1161620. 10.3389/fpsyg.2023.1161620 37275710 10.3389/fpsyg.2023.1161620 PMC10235773

[63] Van der Feltz-Cornelis CM, de Beurs E (2023) The 10-item adverse childhood experience international questionnaire (ace-iq-10): psychometric properties of the Dutch version in two clinical samples. Eur J Psychotraumatol 14:2216623. 10.1080/20008066.2023.2216623 37318018 10.1080/20008066.2023.2216623 PMC10274524

[64] Save the Children Italia (2022) Riprendere insieme a volare. Erickson, Trento

[65] Saunders JB, Aasland OG, Babor TF, de la Fuente JR, Grant M (1993) Development of the alcohol use disorders identification test (AUDIT): WHO collaborative project on early detection of persons with harmful alcohol consumption–II. Addiction 88:791–804. 10.1111/j.1360-0443.1993.tb02093.x 8329970 10.1111/j.1360-0443.1993.tb02093.x

[66] Bush K, Kivlahan DR, McDonell MB, Fihn SD, Bradley KA (1998) The AUDIT alcohol consumption questions (AUDIT-C): an effective brief screening test for problem drinking. Ambulatory care quality improvement project (ACQUIP). Alcohol use disorders identification test. Arch Intern Med 158:1789–1795. 10.1001/archinte.158.16.1789 9738608 10.1001/archinte.158.16.1789

[67] Cortes-Tomas MT, Gimenez-Costa JA, Motos-Selles P, Sancerni-Beitia MD (2016) Different versions of the alcohol use disorders identification test (AUDIT) as screening instruments for underage binge drinking. Drug Alcohol Depend 158:52–59. 10.1016/j.drugalcdep.2015.10.033 26616473 10.1016/j.drugalcdep.2015.10.033

[68] Milne BJ, Caspi A, Crump R, Poulton R, Rutter M, Sears MR, Moffitt TE (2009) The validity of the family history screen for assessing family history of mental disorders. Am J Med Genet B Neuropsychiatr Genet 150B:41–49. 10.1002/ajmg.b.30764 18449865 10.1002/ajmg.b.30764 PMC3750954

[69] Delorme A, Makeig S (2004) Eeglab: an open source toolbox for analysis of single-trial eeg dynamics including independent component analysis. J Neurosci Methods 134:9–21. 10.1016/j.jneumeth.2003.10.009 15102499 10.1016/j.jneumeth.2003.10.009

[70] Ferree TC (2006) Spherical splines and average referencing in scalp electroencephalography. Brain Topogr 19:43–52. 10.1007/s10548-006-0011-0 17019635 10.1007/s10548-006-0011-0

[71] Pascual-Marqui RD, Lehmann D, Koukkou M et al (2011) Assessing interactions in the brain with exact low-resolution electromagnetic tomography. Philos Trans A Math Phys Eng Sci 369:3768–3784. 10.1098/rsta.2011.0081 21893527 10.1098/rsta.2011.0081

[72] Jatoi MA, Kamel N, Malik AS, Faye I (2014) Eeg based brain source localization comparison of sloreta and eloreta. Australas Phys Eng Sci Med 37:713–721. 10.1007/s13246-014-0308-3 25359588 10.1007/s13246-014-0308-3

[73] Asadzadeh S, Yousefi Rezaii T, Beheshti S, Delpak A, Meshgini S (2020) A systematic review of eeg source localization techniques and their applications on diagnosis of brain abnormalities. J Neurosci Methods 339:108740. 10.1016/j.jneumeth.2020.108740 32353472 10.1016/j.jneumeth.2020.108740

[74] Halder T, Talwar S, Jaiswal AK, Banerjee A (2019) Performance evaluation of inverse methods for identification and characterization of oscillatory brain sources: ground truth validation & empirical evidences. bioRxiv. 10.1101/395780

[75] Liu Q, Ganzetti M, Wenderoth N, Mantini D (2018) Detecting large-scale brain networks using eeg: impact of electrode density, head modeling and source localization. Front Neuroinform 12:4. 10.3389/fninf.2018.00004 29551969 10.3389/fninf.2018.00004 PMC5841019

[76] Canuet L, Tellado I, Couceiro V, Fraile C, Fernandez-Novoa L, Ishii R, Takeda M, Cacabelos R (2012) Resting-state network disruption and apoe genotype in Alzheimer’s disease: a lagged functional connectivity study. PLoS ONE 7:e46289. 10.1371/journal.pone.0046289 23050006 10.1371/journal.pone.0046289 PMC3457973

[77] Massullo C, Panno A, Carbone GA, Della Marca G, Farina B, Imperatori C (2022) Need for cognitive closure is associated with different intra-network functional connectivity patterns: a resting state eeg study. Soc Neurosci 17:143–153. 10.1080/17470919.2022.2043432 35167428 10.1080/17470919.2022.2043432

[78] Zinn ML, Zinn MA, Jason LA (2016) Intrinsic functional hypoconnectivity in core neurocognitive networks suggests central nervous system pathology in patients with myalgic encephalomyelitis: a pilot study. Appl Psychophysiol Biofeedback 41:283–300. 10.1007/s10484-016-9331-3 26869373 10.1007/s10484-016-9331-3

[79] Evans ID, Sharpley CF, Bitsika V, Vessey KA, Jesulola E, Agnew LL (2024) Functional network connectivity for components of depression-related psychological fragility. Brain Sci. 10.3390/brainsci14080845 39199536 10.3390/brainsci14080845 PMC11352653

[80] Canuet L, Ishii R, Pascual-Marqui RD et al (2011) Resting-state EEG source localization and functional connectivity in schizophrenia-like psychosis of epilepsy. PLoS ONE 6:e27863. 10.1371/journal.pone.0027863 22125634 10.1371/journal.pone.0027863 PMC3220705

[81] de la Salle S, Choueiry J, Shah D, Bowers H, McIntosh J, Ilivitsky V, Knott V (2016) Effects of ketamine on resting-state EEG activity and their relationship to perceptual/dissociative symptoms in healthy humans. Front Pharmacol 7:348. 10.3389/fphar.2016.00348 27729865 10.3389/fphar.2016.00348 PMC5037139

[82] Kitaura Y, Nishida K, Yoshimura M et al (2017) Functional localization and effective connectivity of cortical theta and alpha oscillatory activity during an attention task. Clin Neurophysiol Pract 2:193–200. 10.1016/j.cnp.2017.09.002 30214995 10.1016/j.cnp.2017.09.002 PMC6123881

[83] Miljevic A, Bailey NW, Vila-Rodriguez F, Herring SE, Fitzgerald PB (2022) Electroencephalographic connectivity: a fundamental guide and checklist for optimal study design and evaluation. Biol Psychiatry Cogn Neurosci Neuroimaging 7:546–554. 10.1016/j.bpsc.2021.10.017 34740847 10.1016/j.bpsc.2021.10.017

[84] Hata M, Kazui H, Tanaka T et al (2016) Functional connectivity assessed by resting state EEG correlates with cognitive decline of Alzheimer’s disease–an eLORETA study. Clin Neurophysiol 127:1269–1278. 10.1016/j.clinph.2015.10.030 26541308 10.1016/j.clinph.2015.10.030

[85] Olbrich S, Tränkner A, Chittka T, Hegerl U, Schönknecht P (2014) Functional connectivity in major depression: increased phase synchronization between frontal cortical EEG-source estimates. Psychiatry Res 222:91–99. 10.1016/j.pscychresns.2014.02.010 24674895 10.1016/j.pscychresns.2014.02.010

[86] Cronbach LJ, Furby L (1970) How we should measure “change”: or should we? Psychol Bull 74:68–80. 10.1037/h0029382

[87] Martin-Garcia O, Blanco I, Sanchez-Lopez A (2024) Do we interpret ambiguity and feel according to how we define ourselves? Relationships between self-perception, interpretation biases, and their role on emotional symptoms. Front Psychiatry 15:1502130. 10.3389/fpsyt.2024.1502130 39758451 10.3389/fpsyt.2024.1502130 PMC11695330

[88] Schneider LH, Hadjistavropoulos HD, Faller YN (2016) Internet-delivered cognitive behaviour therapy for depressive symptoms: an exploratory examination of therapist behaviours and their relationship to outcome and therapeutic alliance. Behav Cogn Psychother 44:625–639. 10.1017/S1352465816000254 27302220 10.1017/S1352465816000254

[89] Geyer RB, Magee JC, Clerkin EM (2023) Anxiety sensitivity and panic symptoms: the moderating influence of distress tolerance. Anxiety Stress Coping 36:618–635. 10.1080/10615806.2022.2146102 36409614 10.1080/10615806.2022.2146102

[90] Frasure-Smith N, Lesperance F, Gravel G, Masson A, Juneau M, Talajic M, Bourassa MG (2000) Social support, depression, and mortality during the first year after myocardial infarction. Circulation 101:1919–1924. 10.1161/01.cir.101.16.1919 10779457 10.1161/01.cir.101.16.1919

[91] McSweeny AJ, Naugle RI, Chelune GJ, Lüders H (1993) “T scores for change”: an illustration of a regression approach to depicting change in clinical neuropsychology. Clin Neuropsychol 7:300–312. 10.1080/13854049308401901

[92] Nichols TE, Holmes AP (2002) Nonparametric permutation tests for functional neuroimaging: a primer with examples. Hum Brain Mapp 15:1–25. 10.1002/hbm.1058 11747097 10.1002/hbm.1058 PMC6871862

[93] Kim HY (2013) Statistical notes for clinical researchers: understanding standard deviations and standard errors. Restor Dent Endod 38:263–265. 10.5395/rde.2013.38.4.263 24303364 10.5395/rde.2013.38.4.263 PMC3843040

[94] Williams MN, Grajales CAG, Kurkiewicz D (2013) Assumptions of multiple regression: correcting two misconceptions. Pract Assess Res Eval 18:11. 10.7275/55hn-wk47

[95] Goncalves SF, Chaplin TM, Turpyn CC, Niehaus CE, Curby TW, Sinha R, Ansell EB (2019) Difficulties in emotion regulation predict depressive symptom trajectory from early to middle adolescence. Child Psychiatry Hum Dev 50:618–630. 10.1007/s10578-019-00867-8 30689145 10.1007/s10578-019-00867-8 PMC6589375

[96] Johnson SL, Porter PA, Modavi K, Dev AS, Pearlstein JG, Timpano KR (2022) Emotion-related impulsivity predicts increased anxiety and depression during the COVID-19 pandemic. J Affect Disord 301:289–299. 10.1016/j.jad.2022.01.037 35026359 10.1016/j.jad.2022.01.037 PMC8747782

[97] Brennan PL, SooHoo S, Lemke S, Schutte KK (2016) Alcohol use predicts 10-year depressive symptom trajectories in the Health and Retirement Study. J Aging Health 28:911–932. 10.1177/0898264315615837 26628481 10.1177/0898264315615837

[98] Powers J, Duffy L, Burns L, Loxton D (2016) Binge drinking and subsequent depressive symptoms in young women in Australia. Drug Alcohol Depend 161:86–94. 10.1016/j.drugalcdep.2016.01.019 26868863 10.1016/j.drugalcdep.2016.01.019

[99] McCabe CJ, Brumback T, Brown SA, Meruelo AD (2023) Assessing cross-lagged associations between depression, anxiety, and binge drinking in the National Consortium on Alcohol and Neurodevelopment in Adolescence (NCANDA) study. Drug Alcohol Depend 243:109761. 10.1016/j.drugalcdep.2022.109761 36621201 10.1016/j.drugalcdep.2022.109761 PMC10122417

[100] Gohari MR, Varatharajan T, Patte KA, MacKillop J, Leatherdale ST (2023) The intersection of internalizing symptoms and alcohol use during the COVID-19 pandemic: a prospective cohort study. Prev Med 166:107381. 10.1016/j.ypmed.2022.107381 36513170 10.1016/j.ypmed.2022.107381 PMC9737513

[101] Uddin LQ (2015) Salience processing and insular cortical function and dysfunction. Nat Rev Neurosci 16:55–61. 10.1038/nrn3857 25406711 10.1038/nrn3857

[102] Schimmelpfennig J, Topczewski J, Zajkowski W, Jankowiak-Siuda K (2023) The role of the salience network in cognitive and affective deficits. Front Hum Neurosci 17:1133367. 10.3389/fnhum.2023.1133367 37020493 10.3389/fnhum.2023.1133367 PMC10067884

[103] Pievani M, Pini L, Ferrari C, Pizzini FB, Boscolo Galazzo I, Cobelli C, Cotelli M, Manenti R, Frisoni GB (2017) Coordinate-based meta-analysis of the default mode and salience network for target identification in non-invasive brain stimulation of Alzheimer’s disease and Behavioral variant frontotemporal dementia networks. J Alzheimers Dis 57:825–843. 10.3233/JAD-161105 28304293 10.3233/JAD-161105

[104] Simmons AN, Stein MB, Strigo IA, Arce E, Hitchcock C, Paulus MP (2011) Anxiety positive subjects show altered processing in the anterior insula during anticipation of negative stimuli. Hum Brain Mapp 32:1836–1846. 10.1002/hbm.21154 21181800 10.1002/hbm.21154 PMC3215249

[105] Enneking V, Klug M, Borgers T et al (2022) Changes in brain function during negative emotion processing in the long-term course of depression. Br J Psychiatry 221:476–484. 10.1192/bjp.2021.223 35082002 10.1192/bjp.2021.223

[106] Lee JY, Jang JH, Choi AR, Chung SJ, Kim B, Park M, Oh S, Jung MH, Choi JS (2021) Neuromodulatory effect of transcranial direct current stimulation on resting-state EEG activity in internet gaming disorder: a randomized, double-blind, sham-controlled parallel group trial. Cereb Cortex Commun 2:tgaa095. 10.1093/texcom/tgaa095 34296150 10.1093/texcom/tgaa095 PMC8152877

[107] Park S, Ryu H, Lee JY, Choi A, Kim DJ, Kim SN, Choi JS (2018) Longitudinal changes in neural connectivity in patients with Internet Gaming Disorder: a resting-state EEG coherence study. Front Psychiatry 9:252. 10.3389/fpsyt.2018.00252 29930524 10.3389/fpsyt.2018.00252 PMC5999751

[108] Liddle EB, Price D, Palaniyappan L, Brookes MJ, Robson SE, Hall EL, Morris PG, Liddle PF (2016) Abnormal salience signaling in schizophrenia: the role of integrative beta oscillations. Hum Brain Mapp 37:1361–1374. 10.1002/hbm.23107 26853904 10.1002/hbm.23107 PMC4790909

[109] De Houwer J, Koster EHW (2023) Attentional biases in anxiety and depression: current status and clinical considerations. World Psychiatry 22:473–474. 10.1002/wps.21117 37713562 10.1002/wps.21117 PMC10503924

[110] Marwood L, Wise T, Perkins AM, Cleare AJ (2018) Meta-analyses of the neural mechanisms and predictors of response to psychotherapy in depression and anxiety. Neurosci Biobehav Rev 95:61–72. 10.1016/j.neubiorev.2018.09.022 30278195 10.1016/j.neubiorev.2018.09.022 PMC6267850

[111] Godfrey KEM, Muthukumaraswamy SD, Stinear CM, Hoeh N (2022) Decreased salience network fMRI functional connectivity following a course of rTMS for treatment-resistant depression. J Affect Disord 300:235–242. 10.1016/j.jad.2021.12.129 34986371 10.1016/j.jad.2021.12.129

[112] Imperatori C, Farina B, Adenzato M, Valenti EM, Murgia C, Marca GD, Brunetti R, Fontana E, Ardito RB (2019) Default mode network alterations in individuals with high-trait-anxiety: an EEG functional connectivity study. J Affect Disord 246:611–618. 10.1016/j.jad.2018.12.071 30605880 10.1016/j.jad.2018.12.071

[113] Wang H, Mou S, Pei X, Zhang X, Shen S, Zhang J, Shen X, Shen Z (2025) The power spectrum and functional connectivity characteristics of resting-state EEG in patients with generalized anxiety disorder. Sci Rep 15:5991. 10.1038/s41598-025-90362-z 39966577 10.1038/s41598-025-90362-z PMC11836123

[114] Dzhebrailova TD, Korobeinikova II, Karatygin NA, Venerina YA, Yantikova EV (2023) Coherence in the EEG theta1 range in the state of relative rest and during testing of attention in subjects with different levels of trait anxiety. Neurosci Behav Physiol 53:1190–1201. 10.1007/s11055-023-01515-4

[115] Chu CS, Lin YY, Huang CC, Chung YA, Park SY, Chang WC, Chang CC, Chang HA (2025) Altered electroencephalography-based source functional connectivity in drug-free patients with major depressive disorder. J Affect Disord 369:1161–1167. 10.1016/j.jad.2024.10.087 39447969 10.1016/j.jad.2024.10.087

[116] Mulert C, Jager L, Schmitt R, Bussfeld P, Pogarell O, Moller HJ, Juckel G, Hegerl U (2004) Integration of fMRI and simultaneous EEG: towards a comprehensive understanding of localization and time-course of brain activity in target detection. Neuroimage 22:83–94. 10.1016/j.neuroimage.2003.10.051 15109999 10.1016/j.neuroimage.2003.10.051

[117] Raichle ME (2011) The restless brain. Brain Connect 1:3–12. 10.1089/brain.2011.0019 22432951 10.1089/brain.2011.0019 PMC3621343

[118] Nagano T, Kurita K, Yoshida T, Matsumoto K, Ota J, Chhatkuli RB, Shimizu E, Hirano Y (2024) Comparison of resting-state functional connectivity between generalized anxiety disorder and social anxiety disorder: differences in the nucleus accumbens and thalamus network. Brain Connect 14:445–456. 10.1089/brain.2024.0034 39135472 10.1089/brain.2024.0034

[119] Greicius MD, Flores BH, Menon V, Glover GH, Solvason HB, Kenna H, Reiss AL, Schatzberg AF (2007) Resting-state functional connectivity in major depression: abnormally increased contributions from subgenual cingulate cortex and thalamus. Biol Psychiatry 62:429–437. 10.1016/j.biopsych.2006.09.020 17210143 10.1016/j.biopsych.2006.09.020 PMC2001244

