# Supplementary material for: Increased beta-band salience network connectivity predicts 12-month longitudinal changes in depressive and anxiety symptom severity in young adults: a resting-state EEG study
Source: Eur Arch Psychiatry Clin Neurosci. 2026 Jun 1;276(5):2681–92. doi: 10.1007/s00406-026-02277-z (PMC13481738; doi:10.1007/s00406-026-02277-z)
Supplement: Supplementary file 1 [file 406_2026_2277_MOESM1_ESM.pdf]

Supplementary Table 1. Completers vs. non-completers between-groups differences.

	**Completers (N= 95)**	**Non-completers (N= 35)**	**Statistic Test**	***p*-value**
Females – N (%)	70 (73.7)	25 (71.4)	CHI^2= .066	.797
Age – M ± SD	21.35 ± 2.65	21.94 ± 2.27	$t\text{-test}_{128}$= -1.180	.240
Tobacco use – N (%)	35 (36.8)	9 (25.7)	CHI^2= 1.415	.234
Students – N (%)	87 (91.6)	32 (91.4)	CHI^2= .001	.978
Bachelor's Degree or higher – N (%)	20 (21.1)	15 (42.9)	CHI^2= 6.181	**.013**
FHS				
FHS-DEP – N (%)	19 (20)	7 (20)		
FHS-ANX – N (%)	13 (13.7)	7 (14.3)	CHI^2= .496	.920
FHS-DEPANX – N (%)	30 (31.6)	5 (14.3)		
AUDIT#3 – M ± SD	.64 ± .89	.43 ± .74	$t\text{-test}_{72.23}$= 1.382	.171
ACE-IQ-10 – M ± SD	1.56 ± 1.57	1.34 ± 1.51	$t\text{-test}_{128}$ =.700	.485
DERS-TOT – M ± SD	40.55 ± 13.12	41.54 ± 12.86	$t\text{-test}_{128}$= -.054	.700
BSI-DEP – M ± SD	1.09 ± .85	.91 ± .74	$t\text{-test}_{128}$= 1.129	.261
BSI-ANX – M ± SD	1.02 ± .80	1.03 ± .76	$t\text{-test}_{128}$= -.079	.938
BSI-DEPANX – M ± SD	2.11 ± 1.51	1.94 ± 1.40	$t\text{-test}_{128}$= .585	.560

Note: In bold significant values
Abbreviation: mean; SD= standard deviation; FHS-DEP= familial risk of depression according to the family history screen; FHS-ANX= familial risk of anxiety according to the family history screen anxiety; FHS-DEPANX= familial risk of depression and anxiety according to the family history screen anxiety; AUDIT#3= item#3 (i.e., binge drinking) of the Alcohol Use Disorders Identification Test; ACE-IQ-10 = 10-item version of the Adverse Childhood Experiences International Questionnaire; DERS= Difficulties in Emotion Regulation Scale; BSI= Brief Symptom Inventory: DEP= depressive symptoms; ANX= anxiety symptoms.

Supplementary Table 2. Anatomical coordinates of the RSNs.

Large-scale network	Region of interest	MNI coordinates		
		x	*y*	*z*
DMN	PCC	0	-52	27
	medial frontal gyrus	-1	54	27
	left AG	-46	-66	30
	right AG	49	-63	33
	left MTG	-61	-24	-9
	right MTG gyrus	58	-24	-9
SN	dACC	0	21	36
	left mFG	-35	45	30
	right mFG	32	45	30
	left INS	-41	3	6
	right INS	41	3	6
	left SMG	-62	-45	30
	right SMG	62	-45	30
CEN	medial frontal gyrus	0	24	46
	superior frontal gyrus	-33	22	53
	left IFG	-44	45	0
	right IFG	44	45	0
	left IPL	-50	-51	45
	right IPL	50	-51	45

Abbreviations: RSNs: resting-state networks; DMN= Default Mode Network; SN= Salience Network; CEN= Central Executive Network; MNI = Montreal Neurological Institute; PCC= posterior cingulate cortex; AG= angular gyrus; MTG= middle temporal gyrus; dACC= dorsal anterior cingulate cortex; mFG= middle frontal gyrus; INS= insula; SMG= supramarginal gyrus; IFG= inferior frontal gyrus; IPL= inferior parietal lobe.

Supplementary Table 3. Values of Pearson's *r* correlation coefficient among RCS-DEPANX values and self-report measures in all sample (N= 95).

	**ACE- IQ-10**	**AUDIT#3^**	**DERS-AW**	**DERS-CLA**	**DERS-GO**	**DERS-IMP^**	**DERS-NAP**	**DERS-STR^**
**RCS-DEPANX**	.053	.279**	.157	.089	.162	.211*	.050	.135

Note: **p*< .05; ***p*< .01; ^: values were square-root transformed prior to analysis.

Abbreviations: RCS-DEPANX= residual change score of depression and anxiety symptoms; ACE-IQ-10 = 10-item version of the Adverse Childhood Experiences International Questionnaire; AUDIT#3= item#3 (binge drinking) of the Alcohol Use Disorders Identification Test; DERS= Difficulties in Emotion Regulation Scale; DERS-AW= awareness; DERS-CLA= clarity; DERS-GO= goal; DERS-IMP= impulse; DERS-NAP= non-acceptance; DERS-STR= strategies.

Supplementary Figure 1. Panel A: within-subject line plot of depression and anxiety (DEPANX) symptoms at Time #1 and Time #2. Panel B: scatterplot of the association between DEPANX symptoms at Time #1 and Time #2. Panel C: scatterplot of the association between DEPANX residual change scores (RCS) and DEPANX symptoms at Time #1. Panel D: scatterplot of the association between RCS-DEPANX and DEPANX symptoms at Time #2.

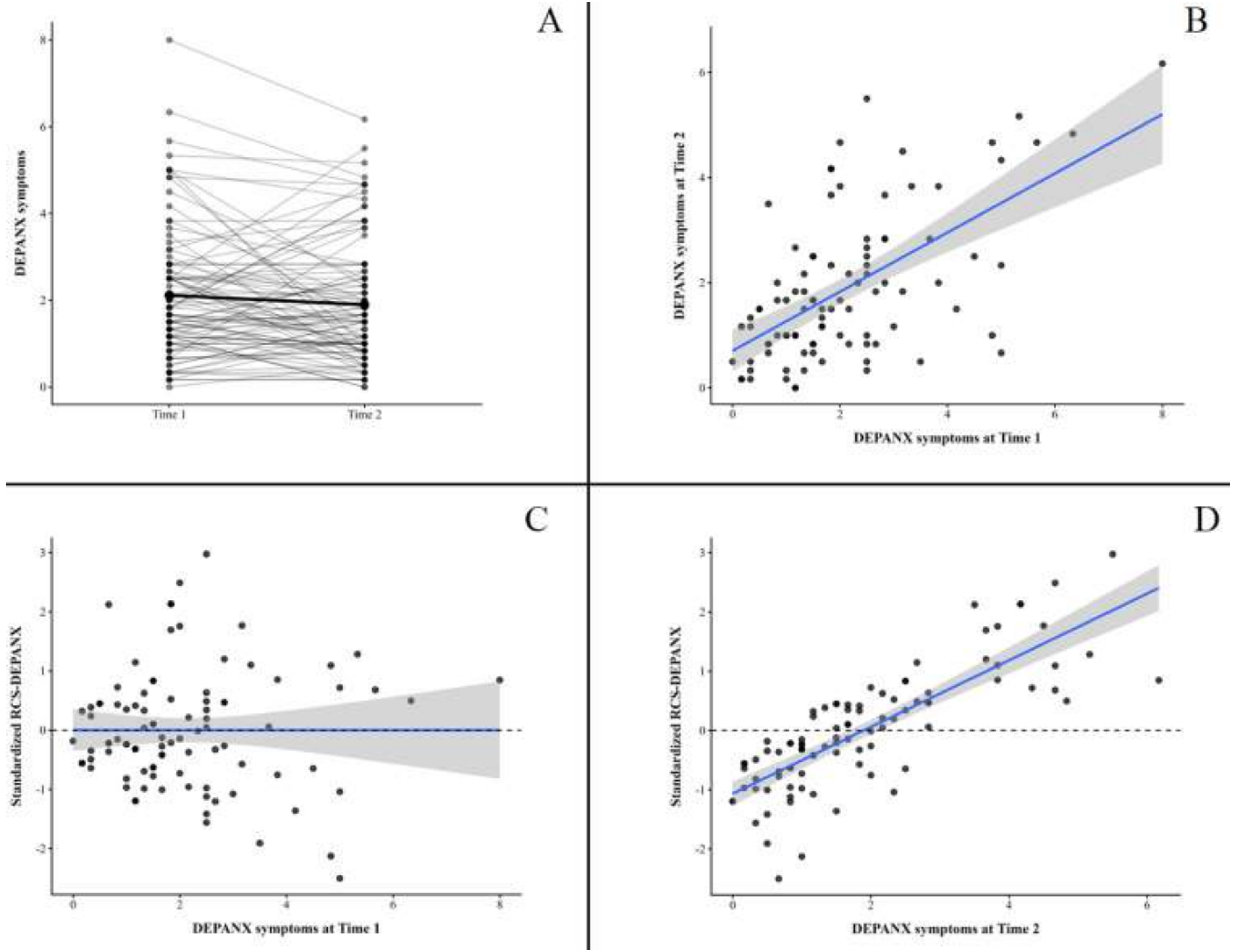

Supplementary Figure 2. The complete correlation matrix between RCS-DEPANX and connectivity values in all interconnected ROIs of the SN, across all frequency bands. Each cell in the matrix represents the Pearson correlation coefficient (r) between the functional connectivity strength of two ROIs and the RCS values. In this analysis, the eLORETA software produced the following thresholds: $r = \pm.381$ corresponding to $p_{adjusted} = .01$, and $r = \pm.343$ corresponding to $p_{adjusted} = .05$. The color bar indicates the magnitude of the correlation coefficients (r), with colors corresponding to the strength of the associations and their significance based on the applied thresholds.
Abbreviations: L= left; R= right; dACC= dorsal anterior cingulate cortex; mFG= middle frontal gyrus; INS= insula; SMG= supramarginal gyrus; SN= Salience Network; RCS-DEPANX= residual change score in depression and anxiety symptoms; ROIs= regions of interest.

DELTA (1–4 Hz)

	dACC	L mFG	R mFG	L INS	R INS	L SMG	R SMG
dACC	-	-.132	-.097	-.112	-.075	-.127	-.237
L mFG	-.132	-	-.104	-.167	-.134	-.201	-.095
R mFG	-.097	-.104	-	-.067	.035	-.097	.012
L INS	-.112	-.167	-.067	-	.071	-.031	-.084
R INS	-.075	-.134	.035	.071	-	-.111	-.054
L SMG	-.127	-.201	-.097	-.031	-.111	-	-.081
R SMG	-.237	-.095	.012	-.084	-.054	-.081	

ALPHA (8–13 Hz)

	dACC	L mFG	R mFG	L INS	R INS	L SMG	R SMG
dACC	-	-.015	.001	.057	-.015	-.010	.069
L mFG	-.015	-	-.023	.032	.225	.103	.143
R mFG	.001	-.023	-	.024	-.018	-.098	.004
L INS	.057	.032	.024	-	.088	.025	.022
R INS	-.015	.225	-.018	.088	-	.239	.106
L SMG	-.010	.103	-.098	.025	.239	-	.099
R SMG	.069	.143	.004	.022	.106	.099	

THETA (4.5–7.5 Hz)

	dACC	L mFG	R mFG	L INS	R INS	L SMG	R SMG
dACC	-	.060	-.012	.107	-.005	-.112	-.006
L mFG	.060	-	.081	.071	.149	-.027	.118
R mFG	-.012	.081	-	.082	.047	-.066	.159
L INS	.107	.071	.082	-	.142	.063	.178
R INS	-.005	.149	.047	.142	-	.011	.014
L SMG	-.112	-.027	-.066	.063	.011	-	.037
R SMG	-.006	.118	.159	.178	.014	.037	

BETA (13.5–30 Hz)

	dACC	L mFG	R mFG	L INS	R INS	L SMG	R SMG
dACC		-.024	.071	.061	.095	.041	.040
L mFG	-.024	-	.072	-.046	.221	.029	.092
R mFG	.071	.072	-	.099	.200	.088	.136
L INS	.061	-.046	.099	-	.371	.075	.144
R INS	.095	.221	.200	.371	-	.133	-.019
L SMG	.041	.029	.088	.075	.133	-	.122
R SMG	.040	.092	.136	.144	-.019	.122	

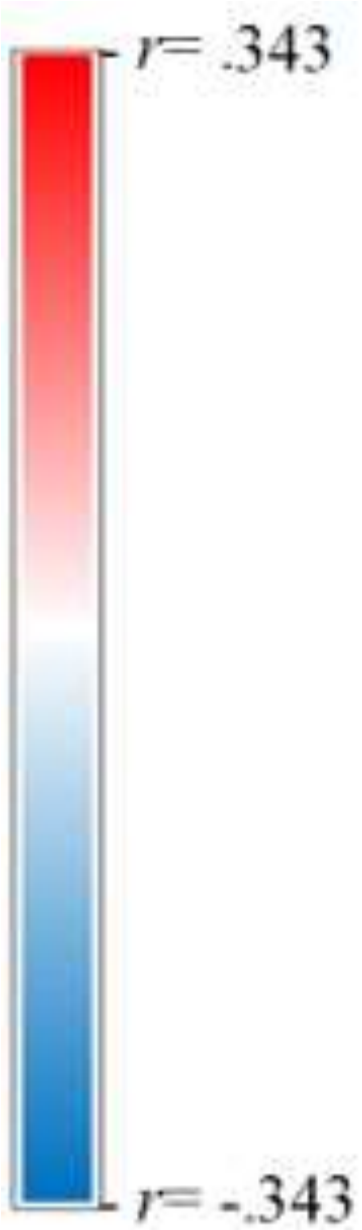

Supplementary Figure 3. The complete correlation matrix between RCS-DEPANX and connectivity values in all interconnected ROIs of the DMN across all frequency bands. Each cell of the matrix shows the Pearson correlation coefficient (r) between the functional connectivity strength of two ROIs and the RCS values. In this analysis, the eLORETA software produced the following thresholds: $r = \pm.376$ corresponding to $p_{adjusted} = .01$, and $r = \pm.332$ corresponding to $p_{adjusted} = .05$. The color bar indicates the magnitude of the correlation coefficients (r), with colors representing the strength of the associations and their significance based on the applied thresholds.

Abbreviations: L= left; R= right; PCC= posterior cingulate cortex; MeFG = medial frontal gyrus; AG= angular gyrus; MTG= middle temporal gyrus; DMN= Default Mode Network; RCS-DEPANX= residual change score in depression and anxiety symptoms; ROIs= regions of interest.

DELTA (1–4 Hz)

	PCC	MeFG	L AG	R AG	L MTG	R MTG
PCC	-	-.171	-.083	-.063	-.097	.025
MeFG	-.171	-	-.194	-.252	-.168	-.148
L AG	-.083	-.194	-	-.085	-.184	-.026
R AG	-.063	-.252	-.085	-	-.111	-.194
L MTG	-.097	-.168	-.184	-.111	-	.022
R MTG	.025	-.148	-.026	-.194	.022	-

ALPHA (8–13 Hz)

	PCC	MeFG	L AG	R AG	L MTG	R MTG
PCC	-	.139	.122	.056	.108	.154
MeFG	.139	-	.050	.086	-.042	.043
L AG	.122	.050	-	.047	.192	.188
R AG	.056	.086	.047	-	.055	.037
L MTG	.108	-.042	.192	.055	-	-.018
R MTG	.154	.043	.188	.037	-.018	-

THETA (4.5–7.5 Hz)

	PCC	MFG	L AG	R AG	L MTG	R MTG
PCC	-	.023	.054	-.001	-.145	-.022
MeFG	.023	-	-.034	.113	.034	.046
L AG	.054	-.034	-	.015	.014	.093
R AG	-.001	.113	.015	-	.013	.135
L MTG	-.145	.034	.014	.013	-	.062
R MTG	-.022	.046	.093	.135	.062	-

BETA (13.5–30 Hz)

	PCC	MeFG	L AG	R AG	L MTG	R MTG
PCC	-	.027	-.130	-.127	-.122	.089
MeFG	.027	-	-.041	-.019	-.002	.098
L AG	-.130	-.041	-	-.166	.061	.043
R AG	-.127	-.019	-.166	-	.023	-.015
L MTG	-.122	-.002	.061	.023	-	.048
R MTG	.089	.098	.043	-.015	.048	-

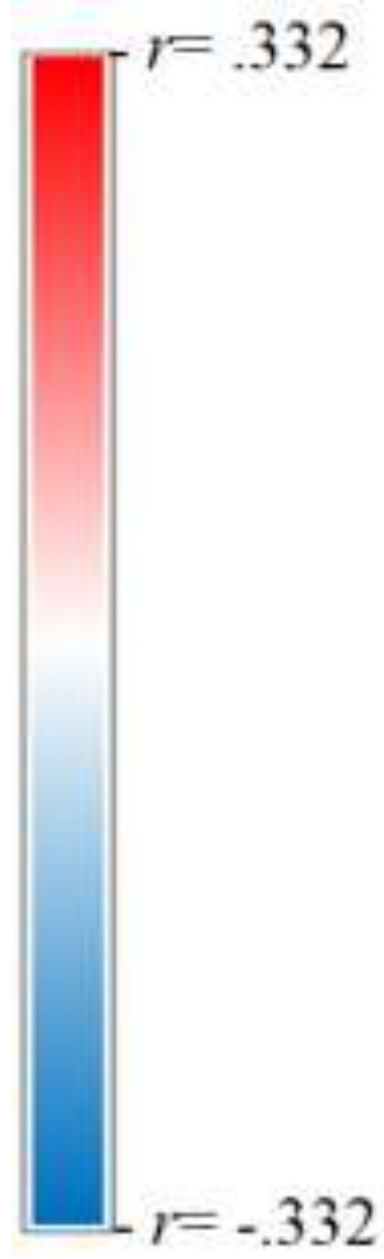

Supplementary Figure 4. The full correlation matrix between RCS-DEPANX and connectivity values in all interconnected ROIs of the CEN, in all frequency bands. Each cell of the matrix represents the Pearson correlation coefficient (r) between the functional connectivity strength of two ROIs and the RCS values. In this analysis, the eLORETA software yielded the following thresholds: $r = \pm.376$ corresponding to $p_{adjusted} = .01$ and $r = \pm.334$ corresponding to $p_{adjusted} = .05$. The color bar indicates the magnitude of the correlation coefficients (r), with colors corresponding to the strength of the associations and their significance based on the applied thresholds.

Abbreviations: L= left; R= right; MeFG = medial frontal gyrus; IFG= inferior frontal gyrus; SFG= superior frontal gyrus; IPL= inferior parietal lobe; CEN= Central Executive Network; RCS-DEPANX= residual change score in depression and anxiety symptoms; ROIs= regions of interest.

DELTA (1–4 Hz)

	MeFG	L SFG	L IFG	R IFG	L IPL	R IPL
MeFG	-	-.079	-.154	-.109	-.016	-.094
L SFG	-.079	-	-.130	-.177	-.173	-.165
L IFG	-.154	-.130	-	-.151	-.065	-.071
R IFG	-.109	-.177	-.151	-	-.025	-.131
L IPL	-.016	-.173	-.065	-.025	-	.003
R IPL	-.094	-.165	-.071	-.131	.003	-

ALPHA (8–13 Hz)

	MeFG	L SFG	L IFG	R IFG	L IPL	R IPL
MeFG	-	.221	.081	.003	.031	.127
L SFG	.221	-	.147	-.031	.057	.137
L IFG	.081	.147	-	-.005	.097	.037
R IFG	.003	-.031	-.005	-	-.048	.003
L IPL	.031	.057	.097	-.048	-	.177
R IPL	.127	.137	.037	.003	.177	-

THETA (4.5–7.5 Hz)

	MeFG	L SFG	L IFG	R IFG	L IPL	R IPL
MeFG	-	-.046	.091	-.030	-.095	.020
L SFG	-.046	-	.032	-.092	-.117	-.005
L IFG	.091	.032	-	-.002	.022	.208
R IFG	-.030	-.092	-.002	-	-.053	.073
L IPL	-.095	-.117	.022	-.053	-	.034
R IPL	.020	-.005	.208	.073	.034	-

BETA (13.5–30 Hz)

	MeFG	L SFG	L IFG	R IFG	L IPL	R IPL
MeFG	-	.049	-.053	.037	-.060	-.043
L SFG	.049	-	.088	-.066	-.082	-.091
L IFG	-.053	.088	-	.016	-.033	.009
R IFG	.037	-.066	.016	-	-.169	-.208
L IPL	-.060	-.082	-.033	-.169	-	-.140
R IPL	-.043	-.091	.009	-.208	-.140	-

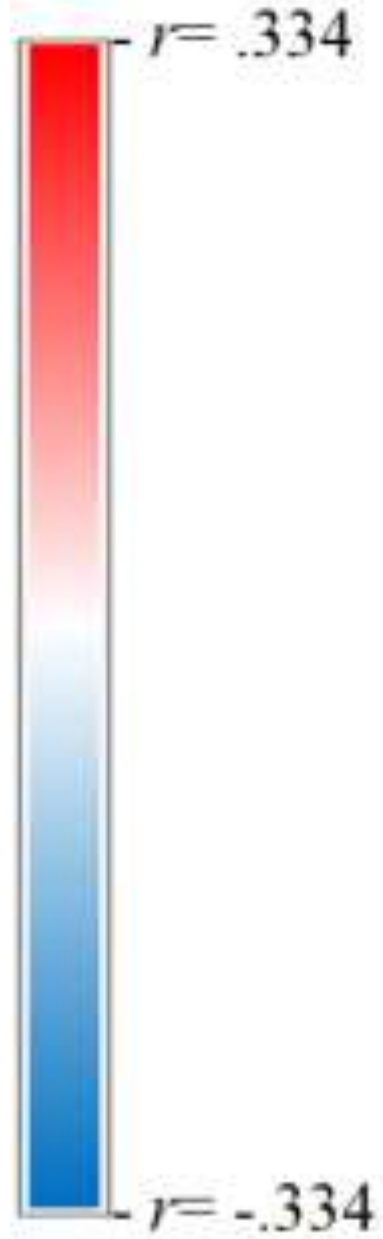

Supplementary Table 4. Unadjusted associations between study variables and residual change score of depression and anxiety symptoms (simple linear regressions).

Predictor	Adjusted R^2	*β*	95% CI	*p*
FHS-DEP	-.010	.027	[-.354; .461]	.795
FHS-ANX	-.010	.033	[-.344; .474]	.753
Sex	-.009	.042	[-.368; .556]	.688
Age	.017	-.165	[-.139; .014]	.110
Student	-.001	.101	[-.371; 1.089]	.332
Bachelor's Degree or higher	-.003	-.087	[-.708; .288]	.404
Psychotherapy in last 12 months	-.010	.035	[-.341; .481]	.736
Tobacco use	.033	.209	[.015; .841]	.042
Cannabis use in the last 12 months	.033	.208	[.014; .898]	.043
AUDIT#3	.092	.319	[.140; .578]	.002
DERS-IMP	.045	.235	[.010; .132]	.022
Bilateral insula beta connectivity	.127	.369	[.176; .557]	<.001

Note: connectivity data are expressed as standardized values; Psychotherapy and Cannabis data use were assessed at Time 2.
Abbreviation: FHS-DEP= familial risk of depression according to the family history screen; FHS-ANX= familial risk of anxiety according to the family history screen anxiety; AUDIT#3= item#3 (i.e., binge drinking) of the Alcohol Use Disorders Identification Test; DERS-IMP= impulse dimension of the Difficulties in Emotion Regulation Scale.

Supplementary Table 5. Associations (i.e., Pearson's *r* and the corresponding *p*-value) among independent variables in the current sample (N= 95).

			1.	2.	3.	4.	5.	6.	7.	8.	9.	10.	11.	12.
**1.**	**Familial risk of depression**	*r*	-											
		p	-											
**2.**	**Familial risk of anxiety**	*r*	.331											
		p	.001											
**3.**	**Female sex**	*r*	.186	.207										
		p	.071	.044										
**4.**	**Age**	*r*	.104	.073	-.139									
		p	.316	.483	.179									
**5.**	**Student**	*r*	-.066	.047	.249	-.421								
		p	.523	.649	.015	.000								
**6.**	**Bachelor's degree or higher**	*r*	.035	-.003	-.160	.550	-.215							
		p	.734	.979	.120	.000	.036							
**7.**	**Psychotherapy in last 12 months**	*r*	-.049	.019	.086	-.010	.188	.071						
		p	.639	.856	.405	.923	.069	.492						
**8.**	**Tobacco use**	*r*	.041	.095	.110	-.109	.153	-.020	.039					
		p	.691	.362	.291	.292	.139	.850	.704					
**9.**	**Cannabis use in the last 12 months**	*r*	.190	.130	.112	-.172	.191	-.096	.111	.438				
		p	.065	.208	.282	.096	.064	.353	.286	.000				
**10.**	**Binge drinking^**	*r*	.156	.075	-.052	-.108	.009	.093	.104	.314	.019			
		p	.131	.471	.615	.299	.931	.368	.316	.002	.852			
**11.**	**DERS-IMP^**	*r*	.119	.216	.154	-.114	.106	-.022	.087	.115	.205	.187		
		p	.253	.035	.136	.272	.305	.835	.400	.266	.046	.070		
**12.**	**Bilateral insula beta connectivity^**	*r*	.065	-.073	.067	-.184	.153	-.012	.085	.081	-.036	.245	.048	
		p	.528	.482	.519	.075	.139	.908	.412	.434	.732	.017	.645	

Note: ^: values were square-root transformed prior to analysis. Abbreviation: DERS-IMP= impulse dimension of the Difficulties in Emotion Regulation Scale.
